# Protective effect of high concentration of BN52021 on retinal contusion in cat eyes

**DOI:** 10.1186/s12886-015-0030-2

**Published:** 2015-05-09

**Authors:** Jin-Feng Huang, Hai-Peng Zhao, Yan-Feng Yang, Hui-Min Huang, Yi Yao, Zhi-Jun Wang

**Affiliations:** Department of Ophthalmology, 307 Hospital, PLA, Beijing, 100071 China; Department of Ophthalmology, CHINA-JAPAN Friendship Hospital, No.2 Yinghua Dongjie, Hepingli, Beijing, 100029 China; Department of Ophthalmology, The General Hospital of PLA, No. 28 Fuxing Road, Beijing, 100853 China

**Keywords:** Retina trauma, BN52021, Electroretinogram, Intraocular pressure, Retinal nerve fiber layer

## Abstract

**Background:**

Blunt injuries/contusion on eyes might cause retina blunt trauma. This study is to evaluate the protective function of BN52021 against retinal trauma.

**Methods:**

A total of 70 cats, 6 months old, were divided into six groups: Group A to E (n = 12) and normal control (N) group (n = 10). The right eyes in Group A to E were contused. All experiments were performed under general anesthetization. Retrobulbar injections of medication in right eyes were performed. Cats were administrated with 0.5 mL of normal saline (NS), dimethyl sulphoxide, 0.2 g/L BN52021, 1 g/L BN52021 and 5 g/L BN52021, respectively. Cats in Group N were administrated with 0.5 mL of NS. Intraocular pressure (IOP), flash electroretinogram (ERG), and retinal nerve fiber layer (RNFL) thickness were measured. Hematoxylin and eosin (HE) staining and transmission electron microscope (TEM) were detected.

**Results:**

No significant difference was observed in IOP levels among groups. Comparing with cats in Group N, those in Group A to E showed significant lower amplitudes of rod a- and b-waves (P < 0.05). Amplitudes of rod a- and b-waves were increased by administration of high concentration of BN52021 (≥1 g/L). Moreover, high concentration of BN52021 decreased the RNFL thickness increased by contusion. Axons in RNFL in Group E arranged neatly at 7 days after modeling.

**Conclusions:**

The degenerated axons caused by contusion were repaired by BN52021. The administration of high concentration of (≥1 g/L) BN52021 could partially repair retinal function in contused cat eyes.

## Background

Contusion/superficial injuries (abrasion) is the most common cause for retina blunt trauma and most of the injuries (accounting for > 25%) are closed globe contusion [1,2]. Blunt injury to eye results in both direct damage at the site of impact tissue and indirect damage to distant intraocular tissues by transmitted forces [3].

Ocular variables as intraocular pressure (IOP) [4,5], electroretinogram (ERG) parameters [6], and retinal nerve fiber layer (RNFL) thickness [7,8] are affected by retina blunt trauma. For instance, Nakayama *et al.* had reported that the amplitudes of the focal macular ERGs after retinal hemorrhages were lower than those of the full-field ERGs [6]. Ahn *et al.* evaluated RNFL thickness using optical coherence tomography (OCT). They determined that RNFL thickness at the second week after trauma was significantly higher than that at the 24th week [7].

*Ginkgo biloba*, which is commonly known as maidenhair tree, is available as a popular herbal supplementary in Asian, European and American countries. Evidences have showed that Ginkgolide B (BN52021, *Ginkgo biloba* extract) has protective function against retinal injuries [9,10]. BN52021 is an antagonist to platelet activating factor (PAF). BN52021 induces irreversible decrease of ERG parameters [9,11,12]. The administration of BN52021 could partially inhibit the irreversible decrease of ERG parameters induced by PAF [9,11]. The protective function of BN52021 on retina has not been systematically reported in cats up to now.

Although there are various differences between human and cat eyes, cats have been serving as models for human vision for a long time [13]. In order to evaluate BN52021’s protective function against retinal blunt trauma, cat models with contused eyes have been established in our study. BN52021 at different concentrations will be administrated to experimental groups. IOP, ERG, and RNFL thickness in cat eyes will be measured. Moreover, hematoxylin and eosin (HE) staining and transmission electron microscope (TEM) will be examined to evaluate the protective function of BN52021 on retina. This study might provide new insights for the management of ocular blunt trauma, harmful to the retina.

## Methods

### Cats and model

The study protocol was approved by the Ethics Committee of the 307th Hospital of Chinese People’s Liberation Army (PLA), affiliated hospital of Military Medical Sciences, Beijing, China. Healthy cats without eye diseases were enrolled in this study. The right cornea surface of all cats was treated with 1% tetracaine (PLA General Hospital) for topical anesthesia. All cats were treated with lower limb general anesthetized with Su-Mian-Xin (Military Veterinary institute of Military Medical sciences, Changchun, China, lot number: 20041010) and Ketamine (Beijing Double-Crane Pharmaceutical Co., Ltd,Lot number:20050202) mixture (1:1, 0.15 mL/kg). Cat was fixed and the right eye was opened with an eye speculum (World Precision Instruments, Sarasota, Florida). A small piece of lens paper was taped on eyes to protect the right cornea. The stainless steel cylinder, weight 0.195 kg, was freely felled from the top of a self-made experimental steel (diameter 2 cm, length 55 cm) with the final impulse of 0.64 kg · m/s to contuse the right eye. After contusion, cats with clear refractive media eyes were selected for further study. Cats with the symptoms such as corneal edema, cataract, hyphema, and vitreous hemorrhage were removed from this study.

Totally, 60 cats (male or female, 6 months old and weighted 2.5 - 3.5 kg) with contused eyes were enrolled in this study. These 60 cats were randomly assigned into 5 groups: Group A (n = 12), B (n = 12), C (n = 12), D (n = 12), and E (n = 12). Another 10 healthy cats without eye diseases were used as normal control (N) group (n = 10). Norfloxacin eye drops (Wuhan Wujing medical Co., Ltd. Wuhan, China) was used to prevent potential eye infections. Cats in Group A to E were administrated with 0.5 mL of normal saline (NS), dimethyl sulphoxide (DMSO, Sigma-Aldrich, Saint Louis, MO, USA), 0.2 g/L BN52021, 1 g/L BN52021 and 5 g/L BN52021 (Tocris Bioscience, Bristol, UK), respectively. Cats in control group were administrated with 0.5 mL of NS. The choice of BN52021’s concentrations was based on the clinical experience and the body weight of cats [14].

### Observation

At 4 h (or 0 h), 1 day, 3 days and 7 days after modeling, three cats in each group were anesthetized for inspections. All inspections were performed under general anesthesia (Su-Mian-Xin and Ketamine mixture). After anesthesia, we performed IOP and ERG measurements immediately. Then the medications of NS, DMSO and BN52021 were retrobulbarily injected into the right eyes. OCT was performed one hour after medication administrations. Then the three cats were euthanized for HE staining and TEM detection. The other cats being left untreated in each group were anesthetized and only administrated with NS, DMSO or BN52021.

### Iop

Methods for IOP measurement had previously been described by Pemp *et al.* [15]. Tropicamide was used to dilate the pupil. Tetracaine eye drops were used for local anesthetics on corneal surface. Measurements of IOP were performed immediately after the anesthesia, with a slit lamp–mounted Goldmann applanation tonometer (Haag-Streit, Bern, Switzerland).

### ERG

Methods for ERG measurement had previously been described by Pawlyk *et al.* [16]. Briefly, cats were dark–adapted at constant temperature for 1 h. Tropicamide was used to dilate the pupil and all cats were anesthetized. The observations were performed by one of the authors. Rod-dominated responses were elicited in dark with 10-μs flashes of white light (3.556 × 10^−2^ cd*s/m^2^) presented in a Ganzfeld dome. Maximum analysis time was 250 ms with the same flashes presented at 1 Hz. The retinal signals were band-pass filtered between 0.1 and 75 Hz, amplified 5,000-fold and averaged (5 repetitions). The amplitudes and latency periods of a- and b-waves, as well as the oscillograms after dark adaptation were recorded.

### OCT

Methods for OCT measurement had previously been described by Ko *et al.* [17], with a Zeiss-humphery OCT 3.0 (Zeiss Humphery Instruments, Dublin, California, USA). RNFL thickness in macula was quantified and averaged (5 repetitions at different point).

### HE staining

After the measurements of IOP, ERG and OCT, three cats in each group were euthanized for HE staining and TEM detection at each time point. Methods for the HE had previously been described by Assaf *et al.* [18]. In brief, the optic nerve near the eyeball were cut, fixed in 4% glutaraldehyde for 48 h, dehydrated with a graded series of ethanol (2 h for each grade), transparented in dimethylbenzene and embedded in paraffin. Sections were cut by a microtome (Leica, Tokyo, Japan) and collected over polylysine treated slides. The slides were dewaxed, dehydrated, incubated with hematoxylin stain for 5 min, differentiated with 1% hydrochloric acid alcohol for 30 s, backed to blue with 1% ammonium hydroxide and stained with 0.5% eosin solution for 5 min. After the final dehydration and transparention, slides were sealed with neutralresinsize and examined using an optical microscope (Leica DMLS; Leica Microsystems Inc., Depew, New York, USA).

### TEM

We performed the TEM detection according to the methods described by Wang *et al.* [19]. Three specimens in each group were cut into small pieces, 1 mm × 1 mm × 1 mm. These pieces were washed with sodium cacodylate buffer, fixed in 2.5% glutaraldehyde, dehydrated with a graded series of ethanol, sectioned with an ultramicrotome (Leica, Japan) and stained with saturated uranium acetate. TEM detections were performed by one of the authors using a JEM 100CX electron microscope (JEOL, Tokyo, Japan).

### Statistical analysis

Each experiment was performed in triplicates, and the average values were calculated. SPSS 17.0 statistical software was used for data variance analysis. Experimental data were expressed as mean ± standard deviation (SD). Unequal variances will be corrected with Welch. Comparison between two groups involved a paired sample t-test and comparison among three or more groups, a repeated measures one-way ANOVA and post hoc test. P < 0.05 was set as the threshold for significant difference.

## Results

### IOP

**Figure 1:**
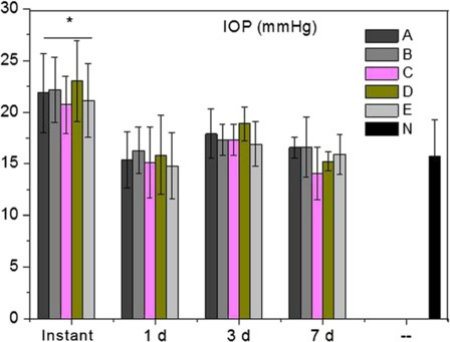
Mean intraocular pressure. * indicates P < 0.05 compared with other groups at 1 day, 3 days and 7 days after modeling.

As shown in Figure 1, IOP levels at the onset after modeling were significantly higher than those at 1 day, 3 days and 7 days after modeling (P < 0.05). The IOP levels in experimental groups were significantly decreased at 1 day, 3 days and 7 days after modeling. However, no significant difference was observed in IOP levels among groups at the same experimental point (Figure 1).

### ERG

**Figure 2:**
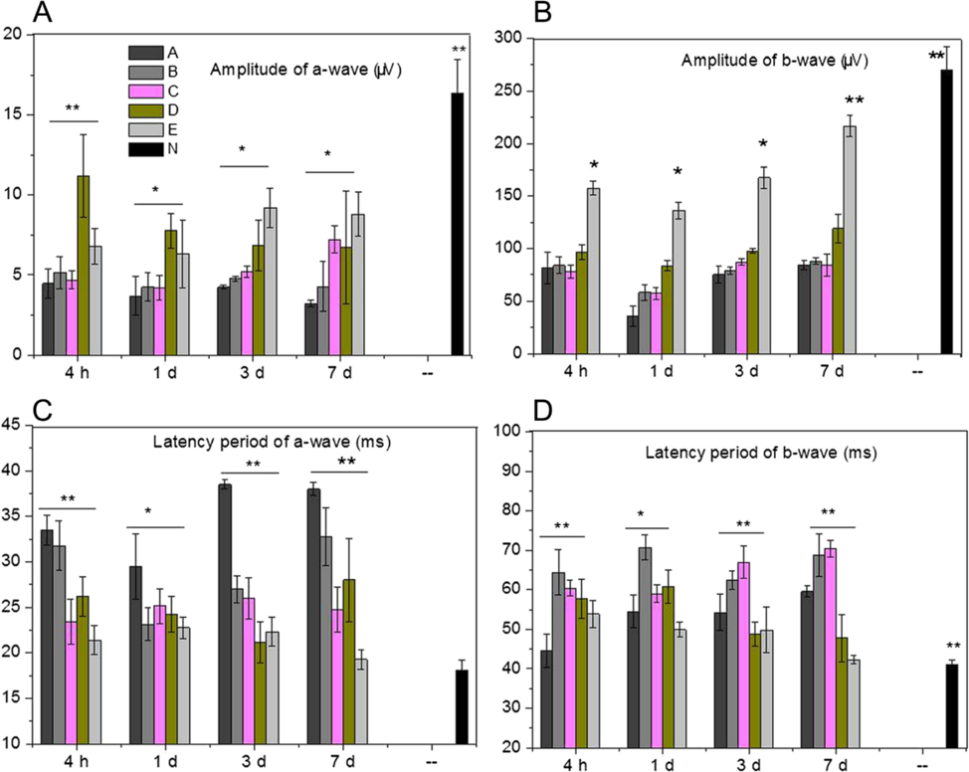
The electroretinogram (ERG) amplitudes and latency periods of a- and b-waves. **A**, ERG amplitude of a-wave; **B**, ERG amplitude of b-wave; **C**, ERG latency period of a-wave; **D**, ERG latency period of b-wave. * and ** indicates P < 0.05 and P < 0.01 compared with the other groups at the same treatment time point, respectively.

Comparing with Group N, the amplitudes of a- and b-waves in experimental groups were significantly decreased (P < 0.05, Figure 2A,B). The amplitude of a-wave in Group A was lower than those in other groups (P > 0.05). The amplitude of a-wave in Group D showed a significant decrease during the experimental period (Figure 2A). However, the amplitudes of a-wave in Group E at 3 days and 7 days after modeling were significantly higher than those in other groups (P < 0.05, Figure 2B). Moreover, the amplitude of b-wave in Group E was higher than those in other groups (P < 0.0.5).

The cats in Group A had significant longer latency periods of a-wave than other groups (P < 0.01, Figure 2C). The latency period of b-wave in Group E was shorter than those in other groups (P < 0.05, Figure 2D). Moreover, the administration of BN52021 significantly decreased the latency period of a- and b-waves in Group E (P < 0.05).

**Figure 3:**
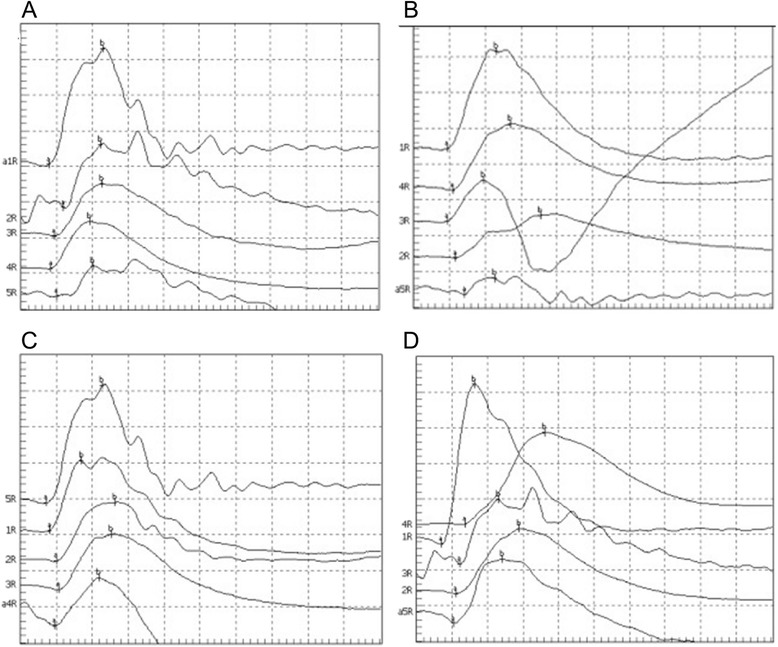
Tracings of oscillograhic records of cat retinas after dark adaptation. **A - D**, represent 4 inspection time points: 4 h, 1 day, 3 days and 7 days after modeling. From up to down, oscillograms for cats in Group E, D, C, B, and A, respectively. The stimulus duration is 250 ms.

For the oscillograms measurement, the cats in Group E showed highest levels along with the experimental duration. Oscillograms of b-wave in Group E showed an obvious increment than those in other groups (Figure 3).

### OCT

**Figure 4:**
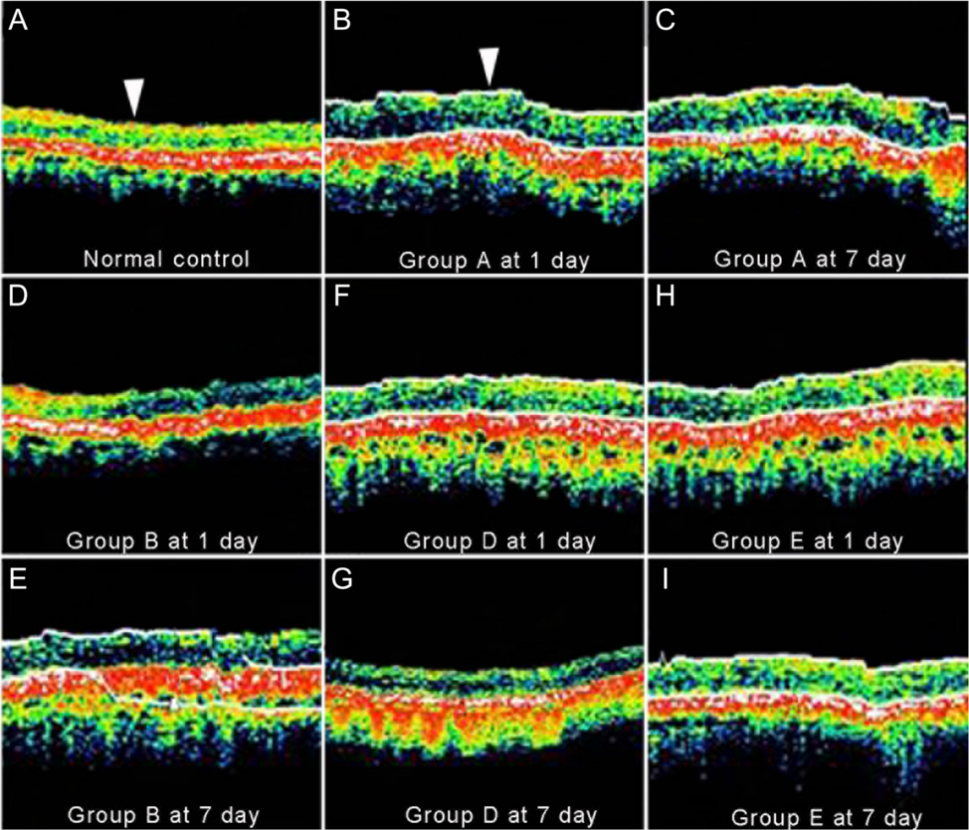
Optical coherence tomography (OCT) results of the retinal nerve fiber layer (RNFL) during the experimental periods. **A**, OCT result of the normal RNFL; **B** and **C**, OCT results of the RNFL in Group A at 1 day and 7 days after modeling with 0.5 mL of normal saline administration. An obvious swelling was observed in RNFL; **D** and **E**, OCT results of the RNFL in Group B at 1 day and 7 days after modeling, with 0.5 mL of DMSO administration; **F** and **G**, OCT results of the RNFL in Group D at 1 day and 7 days after modeling, with 0.5 mL of 1 g/L BN52021 administration; **H** and **I**, OCT results of the RNFL in Group E at 1 day and 7 days after modeling, with 0.5 mL of 5 g/L BN52021 administration. Comparing with the normal RNFL, all RNFLs in the experimental groups were swelling to some extent. For **A**, **D** and **G**, bar = 150 μm, for **B**, **C**, **E**, **F**, **H** and **I**, bar = 175 μm.

**Figure 5:**
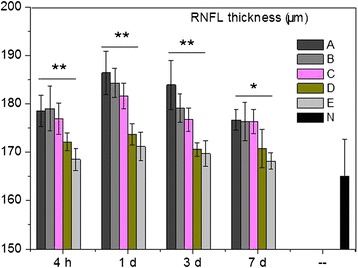
Results of retinal nerve fiber layer (RNFL) thickness examination. Significant differences could be seen among the groups. The administrations of middle concentration (1 g/L, 0.5 mL) and high concentration (5 g/L, 0.5 mL) of BN52021 significantly and very significantly decreased the RNFL thickness. * and ** represents significant level at P < 0.05 and P < 0.01, respectively.

After modeling, RNFL thickness in Group A was significantly higher than that in control (Figure 4A-C). DMSO administration slightly decreased RNFL thickness (Figure 4D,E). Moreover, RNFL thickness decreased along with the increment of BN52021 concentration and handling time (Figure 4E-I). As shown in Figure 4 and Figure 5, RNFL thicknesses in Group D and E were significantly decreased than those in other groups (P < 0.05). RNFL thickness in Group E at 7 days after modeling was at the same level as that in control (Figure 5).

### HE staining

**Figure 6:**
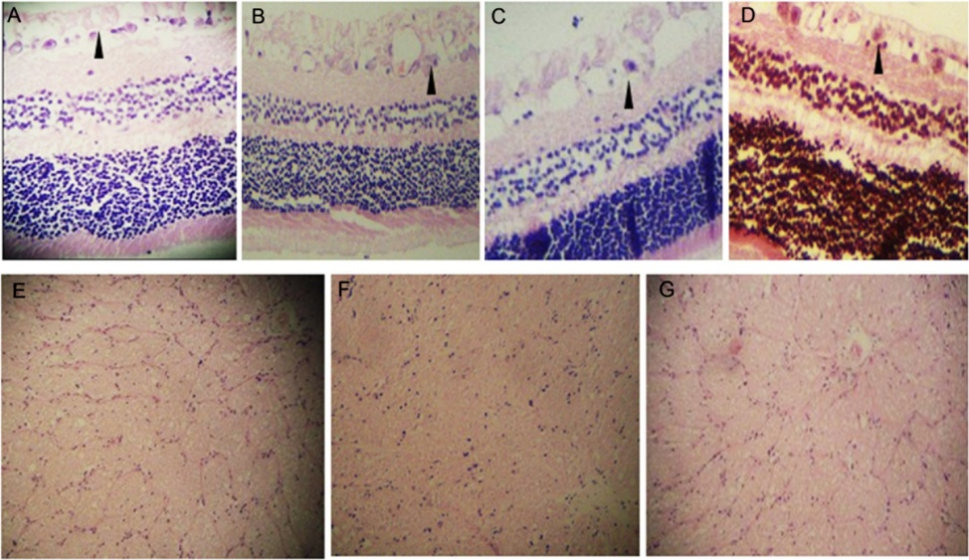
Hematoxylin and eosin (HE) staining results of the cell layers in retina and anterior optic nerves. **A**, HE staining result of the cell layers in normal retina; **B** and **C**, HE staining results of retina in Group A and B administrated with normal saline and DMSO at 1 day after modeling, respectively; **D**, HE staining result of retina in Group E administrated with high concentration (5 g/L, 0.5 mL) of BN52021 at 7 days after modeling. **E** to **G**, HE staining results of anterior optic nerves in normal retina **(E)** and contusion retina at 4 h **(F)** and 7 days **(G)** after modeling. The arrows in Figure **A-D** indicate the ganglionic layers in the RNFL. **A** to **D**, original magnification × 400. **E** to **G**, original magnification × 100.

As shown in Figure 6, all cell layers of retina in control were clear and neatly arranged (Figure 6A). Cells were disorganized after modeling. Obvious swellings in RNFL and nucleus disintegration in ganglionic layers were observed (Figure 6B). RNFL swelling was slightly reduced by DMSO administration (Figure 6C). The administration of high concentration of BN52021 significantly attenuated RNFL swelling. Ganglionic layers were neatly arranged in Group E (Figure 6D). Moreover, the anterior optic nerves were not damaged by contusions (Figure 6E - G). The nerve fibers were neatly arranged without difference between control and Group A (data in Group B-E were not shown).

### TEM

**Figure 7:**
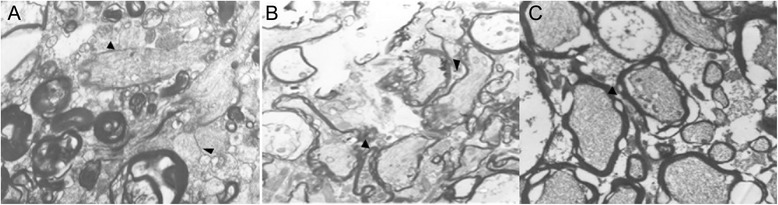
Transmission electron microscope (TEM) results of the anterior optic nerves in cat eyes. **A**, anterior optic nerves in normal cat eyes; **B**, anterior optic nerves in contused cat eyes; **C**, anterior optic nerves in cat eyes administrated with high concentration (5 g/L, 0.5 mL) of BN52021. Black arrows indicate the space between axons. **A**, original magnification × 10000. **B** and **C**, original magnification × 4000.

Figure 7 showed the TEM results of the anterior optic nerves. Myelin sheath layers were neatly and tightly arranged in control cats (Figure 7A). Axons were degenerated and filled with vacuoles, and onion skin lesions were observed after modeling (Figure 7B). After being administrated with high concentration of BN52021, the lesions in Group E were significantly reduced and the spaces between axons were reduced (Figure 7C).

## Discussion

Thierry *et al.* have reported that the presence of tritiated PAF ([^3^H]PAF) specific binding sites which possess a Kd of 2.9 ± 0.4 nM and Bmax of 0.85 ± 0.16 pmol/mg protein in membrane of albino rats retina. The binding of [^3^H]PAF to these specific sites are saturable, specific, time-dependent and reversible and are considered to be related to PAF-induced disturbances of ERG b-wave [20].

As previously reported, BN52021’s protective effect on retinal is dose-dependent. High concentration of BN52021 (2 × 10^−5^ M) could partially inhibit the irreversible decrease of ERG b-wave amplitude induced by PAF-acether [9]. Our findings suggest that high concentration of BN52021 (≥1 g/L) could partially restore the destroyed cat retina by decreasing the ERG amplitudes of a- and b-waves. This result is inconsistent with previous data showing that BN52021 administration could induce the inhibition of b-wave amplitude [9,11]. These results demonstrate again that BN52021 could partially inhibit the irreversible decrease of the ERG b-wave amplitude induced by PAF-acether [9,11,12]. Moreover, the oscillograms can be considered to be reflection of retinal function [21]. In this study, the b-wave oscillograms in the group treated with high concentration of BN52021 showed obvious increment comparing with those in other groups treated with lower concentration of BN52021. These results suggest the retina as well as hyperpolarizing cone bipolar cells or horizontal cells might have been harmed by contusion. Retinal function might have been partially restored by BN52021 administrations.

Retinopathy is usually caused by injury and inflammatory on eyes, so did brain diseases as head trauma [22,23]. Inflammation serves as an important factor for the development of retinopathy [24]. Diseases as diabetes cause metabolic and physiologic abnormalities in retina, including the upregulation of iNOS, NF-kB and the increment of leukostasis and PAF. However, BN52021 could attenuate tissue damage caused by these abnormalities [10], including suppressing PAF induced paw swelling [25].

Evidences have suggested that BN52021 not only act as a PAF antagonist but also serve as a free radical scavenger for reactive oxygen species [26,27]. Thus, BN52021 could inhibit oxidation and decomposition of low-density lipoprotein [28]. BN52021 inhibits the increase of IOP and the damage to retina [29-32]. In this current study, administration of BN52021 reduced IOP levels and RNFL thickness, as well as attenuated the damage to RNFL. These results demonstrat that BN52021’s protective effect on retinal function might relate to those inflammatory responses.

Nonetheless, there are also some limitations existing in this study. It might be more serious to perform the experiment with smaller grades of BN52021 concentration. It is more helpful to perform experiments on inflammation factors for investigation of BN52021’s protective effect. In our study, the ERG amplitudes of a- and b-waves reduced by contusion were triggered by BN52021 administration. RNFL swelling and thickness increment were attenuated by BN52021 administration. Moreover, BN52021 administration significantly reduced the lesions in degenerated axons of anterior optic nerves. These suggested that high concentration of BN52021 (≥1 g/L) might have significant protective effects on retinal function of cat eyes. These hypotheses would be proved by more than one experiment on the inflammatory function of BN52021.

## Conclusions

In conclusion, high concentration of BN52021 administration (≥1 g/L) could significantly increase amplitudes of a- and b-waves and reduce latency periods of a- and b- waves which were influenced by contusion. Moreover, BN52021 could decrease the RNFL thickness increased by contusion. This study demonstrates that BN52021 is a protective drug for retina function in cat eyes and might provide us with new insights for the management of ocular blunt trauma.

## Abbreviations

ERG: Electroretinogram
HE: Hematoxylin and eosin
IOP: Intraocular pressure
OCT: Optical coherence tomography
PAF: Platelet activating factor
PLA: People’s Liberation Army
RNFL: Retinal nerve fiber layer
TEM: Transmission electron microscope
